# Risk stratification system of gastrointestinal stromal tumors under EUS elastography based on artificial intelligence

**DOI:** 10.1097/eus.0000000000000136

**Published:** 2025-09-05

**Authors:** Chenxia Zhang, Wei Tan, Xun Li, Xiao Tao, Bing Xiao, Wei Zhou, Honggang Yu

**Affiliations:** Department of Gastroenterology, Renmin Hospital of Wuhan University; Hubei Provincial Clinical Research Center for Digestive Disease Minimally Invasive Incision, Renmin Hospital of Wuhan University; Key Laboratory of Hubei Province for Digestive System Disease, Renmin Hospital of Wuhan University, Wuhan, Hubei Province, China.

**Keywords:** Gastrointestinal stromal tumors, EUS elastography, Risk stratification, Artificial intelligence

## Abstract

**Background and Objectives:**

Gastrointestinal stromal tumors (GISTs) are tumors with malignant potential, and preoperative risk stratification is critical for clinical management. Endoscopic ultrasound elastography (EUS-E) can assess tissue stiffness and may assist in evaluating the malignant potential of GISTs. However, similar studies have not been conducted, and current elasticity evaluation methods are still highly influenced by operator's subjectivity. An effective and objective tool is needed to aid in the risk stratification of GISTs under EUS-E.

**Methods:**

One hundred eighty-nine patients with submucosal tumors (SMTs) who underwent EUS-E from January 2018 to August 2024 were retrospectively collected, of which 110 cases were GISTs. A total of 2625 EUS B-mode images were collected to construct the classification and segmentation model to distinguish GISTs from non-GISTs and to segment the lesion areas of GIST automatically. The elasticity value (EUS-E-AI) of the lesion area was extracted based on the color features of the elastography images. We evaluated the diagnostic performance of this system in distinguishing GISTs from other SMTs, as well as its ability to stratify GISTs based on their malignant potential.

**Results:**

The accuracy of the classification model and the Dice coefficient of the segmentation model were 95.8% and 0.967, respectively. The EUS-E-AI value in the low-risk malignancy group (0.268 [IQR, 0.243–0.333]) was significantly higher than that in the high-risk malignancy group (0.186 [IQR, 0.176–0.199], *P* < 0.001). A cutoff value of 0.224 for the EUS-E-AI was found to effectively differentiate the low-risk from the high-risk group, with an accuracy of 92.6% (95% CI, 89.1–96.1). These findings were also confirmed in small GISTs.

**Conclusion:**

We developed an AI-based system and elasticity indicator for the accurate and objective identification and risk stratification of GISTs using EUS.

## INTRODUCTION

Gastrointestinal stromal tumors (GISTs) are the most common mesenchymal tumors in the digestive system, with an annual incidence of 4 to 20 cases per million people.^[1]^ These tumors primarily arise from interstitial cells of Cajal or their precursors and can occur anywhere along the gastrointestinal tract, with 50%–60% found in the stomach.^[2]^ GISTs have complex biological behaviors, varying from incidental small tumors to highly aggressive sarcomas.^[3]^ Curative treatment options for GISTs include surgical resection, which remains the standard of care for tumors larger than 2 cm.^[1,4]^ For small GISTs, minimally invasive endoscopic techniques such as submucosal tunneling endoscopic resection (STER) and endoscopic submucosal dissection (ESD) have also been shown to achieve complete resection in experienced centers.^[5]^ However, some high-risk GIST patients continue to face the risk of tumor recurrence and metastasis even after complete tumor resection.^[6]^ With the advent of neoadjuvant targeted therapies, early identify high-risk malignancy groups and implement preoperative treatment may facilitate achieving R0 resection, which is crucial for improving patient outcomes.^[1,7]^ Therefore, accurate preoperative risk stratification of GISTs is essential for guiding clinical decisions and assessing patient prognosis.

Risk stratification of GISTs is typically based on the National Institutes of Health (NIH) consensus classification system, which categorizes tumors into 4 risk levels (high, intermediate, low, and very low risk) according to factors such as tumor size and mitotic count.^[3,8]^ However, preoperative samples are often obtained via EUS–guided fine-needle aspiration/biopsy (EUS-FNA/B) or computed tomography (CT)–guided percutaneous approach.^[1,9]^ Due to tumor heterogeneity and the inherent limitations of the biopsy specimen, these methods may not fully capture the overall characteristics of the tumor.^[10]^ Moreover, as GISTs tend to be friable, biopsy procedures can lead to complications such as bleeding, rupture, or intraperitoneal spread.^[11]^ Thus, developing a noninvasive, reproducible, and accurate preoperative risk assessment method for GISTs is of significant clinical value.

Although CT and magnetic resonance imaging (MRI) play a pivotal role in diagnosing GISTs, the information they provide is often limited. EUS, with its advantage of wall-adherent scanning, has become the most accurate method for assessing submucosal tumors (SMTs).^[12]^ With advancements in EUS technology, EUS elastography (EUS-E) has gained widespread clinical use for evaluating tissue stiffness, allowing for the differentiation between benign and malignant lesions.^[13–15]^ The application of EUS-E has the potential to improve both the accuracy of GIST differentiation and risk stratification.^[16]^ However, the interpretation of elastography remains challenging, as it often requires manual circle selection of the lesion area by the operator to obtain elasticity values. This reliance on lesion selection and the subjectivity of elasticity assessment can impact the overall diagnostic accuracy.

As a result, there is a growing demand for more objective and accurate methods to assess lesion elasticity and evaluate the malignant potential of GISTs. In recent years, artificial intelligence (AI) has been increasingly applied in medical imaging.^[17]^ Although deep learning has been used to differentiate GISTs from other SMTs on EUS, no studies have yet explored the potential of EUS-E in risk stratification for GISTs.^[18–20]^ To address this gap, we have developed an AI-based risk stratification system for GISTs using EUS-E. This innovative approach employs AI-driven automatic extraction of elasticity (EUS-E-AI) to classify GISTs according to their risk levels. Additionally, we have established threshold values for elasticity measurements, enabling automated risk stratification of GISTs and providing guidance for EUS-FNA/B based on the risk distribution within the lesion.

## PATIENTS and METHODS

### Patients

Patients diagnosed as SMTs through EUS-FNA/B or endoscopic/surgical resection at Renmin Hospital of Wuhan University between January 2018 and August 2024 were retrospectively collected. The inclusion criteria were as follows: (1) age >18 years (2) pathological confirmation of SMTs (e.g., GISTs, leiomyomas, neuroendocrine tumors, ectopic pancreas), and (3) EUS-E performed within 2 weeks prior to operation. EUS B-mode and EUS-E images were obtained for all patients and stored in JPEG format. Poor-quality images due to air interference, blurring, or defocus were excluded. The study was approved by the Ethics Committee of Renmin Hospital of Wuhan University. Given its retrospective design, the requirement for written informed consent was waived.

### EUS procedure

EUS was performed using an Olympus EU-ME2 processor (Olympus Corporation, Tokyo, Japan) and adapted endoscopes. All procedures were conducted by operators with over 5 years of experience in EUS. Initially, a conventional B-mode module was used to scan the lesions. Once the SMTs were identified, the operator switched to the elastography module to perform EUS-E on the region of interest (ROI). The elastography technique used in this study was real-time strain elastography, a qualitative method that visualizes relative tissue stiffness based on deformation under manual compression. During this procedure, the EUS-E frequency was set to 7.5 MHz, and the elastography image was overlaid onto the traditional B-mode image. The resulting color map represented tissue stiffness, with softer tissue (larger strain) shown in red and harder tissue (higher stiffness) shown in blue, aiding in the differentiation of benign and malignant lesions [Figure S1, http://links.lww.com/ENUS/A382].

### EUS-E-AI

**Figure 1:**
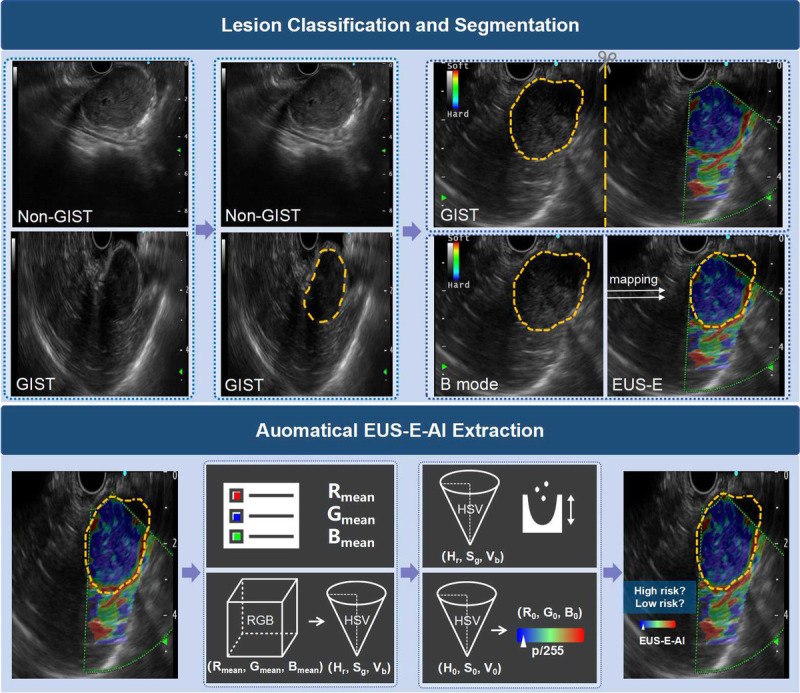
The framework of risk stratification system.

As shown in Figure 1, 4 steps were involved to obtain EUS-E-AI. First, 2 deep convolutional neural network (DCNN) models were used for lesion classification and segmentation. DCNN1 identifies SMTs in EUS B-mode images and classifies them into GISTs and non-GISTs, thereby activating downstream models. DCNN2, using EUS B-mode images, outlines the boundaries of GIST lesions and feeds them into module 3. Subsequently, module 3 maps the segmentation marks from the EUS B-mode image to the corresponding EUS-E image. Finally, module 4 extracts the color features from the EUS-E image in the lesion area to calculate elasticity, EUS-E-AI, for that region.

We employed ResNet for image classification and Unet++ for image segmentation in DCNN1 and DCNN2. Images from the same patient were not included in both the training and test sets. Specifically, we collected 2625 EUS B-mode images from 189 SMT patients (1571 GIST images and 1054 from other SMTs) to train and validate DCNN1 in a ratio of 9:1. Subsequently, an expert endoscopist with over 5 years of EUS experience outlined the boundaries of the lesion in 1571 EUS B-mode images from 110 GIST patients to train and test DCNN2.

For lesion segmentation in module 3, we collected 213 EUS-E images from 84 GIST patients and followed these steps: (1) divide each EUS-E image into 2 panels using the middle marker line, with the left panel displaying the B mode image and the right panel displaying the elastography image, and (2) through symmetry processing, map the segmentation markers from the B mode image in DCNN2 to the elastography image in the right panel. After obtaining the lesion segmentation markers from the EUS-E image, module 4 extracted the pixel values (RGB channel values) from all color pixels in the lesion area. To ensure the reliability of elasticity assessment, segmentation and elasticity extraction were confined to the solid components of the lesion. The average pixel value was then calculated by averaging the pixel values across all extracted data, resulting in a pixel coordinate composed of the average values for the R, G, and B channels.

Further, the R, G, and B channels were used to define an RGB coordinate system, and the average pixel value was converted into the HSV color space to more accurately reflect its position on the color scale, denoted as (H_r_, S_g_, V_b_). Then, using the minimum distance approach, the closest intermediate coordinates (H_0_, S_0_, V_0_) in the HSV color space were identified. Finally, through the conversion between the RGB and HSV coordinate systems, the corresponding coordinates in the RGB system (R_0_, G_0_, B_0_) were determined to define the EUS-E-AI of the lesion area. Specifically, the position of (R_0_, G_0_, B_0_) within the RGB color map (from blue: 0 to red: 255) was calculated and converted into an elasticity value ranging from 0 to 1, referred to as EUS-E-AI. The EUS-E-AI reflects the tissue's stiffness, with smaller values indicating stiffer tissue. Further details on the model construction can be found in the Supplementary Materials, http://links.lww.com/ENUS/A382.

### Risk stratification

To evaluate malignant potential, we classified the NIH risk categories into “low-risk malignancy” (very low-risk and low-risk) and “high-risk malignancy” (intermediate-risk and high-risk) groups. Details of the NIH consensus classification are shown in Table S1, http://links.lww.com/ENUS/A382. The EUS-E-AI values were compared between the low-risk and high-risk malignancy groups. The Youden index was applied to the receiver operating characteristic (ROC) curve to determine the optimal cutoff point for distinguishing high-risk from low-risk malignant potential GIST patients.

### Statistical analysis

Normally distributed continuous variables were presented as mean ± standard deviation (SD), whereas nonnormally distributed data are expressed as median with interquartile range (IQR). Comparisons between the 2 groups were conducted using the *t* test or Mann-Whitney *U* test. Categorical variables were described as frequency (percentage), and intergroup comparisons were made using the *χ*^2^ test or Fisher's exact test. Spearman's rank correlation analysis was performed to evaluate the correlation between EUS-E-AI, GIST risk stratification, and the pathological mitotic count. Logistic regression analysis was employed to assess the effect of patient age, gender, lesion size, lesion location, and EUS-E-AI on risk stratification. All significance tests were 2-tailed, and a *P* value of less than 0.05 was considered statistically significant. The analyses were conducted using SPSS 25 (IBM, Chicago, IL, USA).

## RESULT

### Patient characteristics

From January 2018 to August 2024, a total of 189 pathologically diagnosed SMT patients underwent EUS-E (110 GISTs and 79 non-GISTs). Among the 110 GIST patients, 19 lacked the information about risk stratification in postoperative pathological reports, 5 had no available EUS-E images, and 2 had poor-quality images. The low-risk malignancy group included 46 GIST patients (15 with very low risk and 31 with low risk according to the NIH risk classification), whereas the high-risk malignancy group consisted of 38 patients (29 with intermediate risk and 9 with high risk according to the NIH risk classification) [Figure S2, http://links.lww.com/ENUS/A382]. Table 1 presents the baseline characteristics of all patients. The average age was 57.6 ± 11.57 years, with 92 male patients (48.7%). Among the patients, 108 (57.1%) had lesions larger than 2 cm, and GISTs in the low-risk malignancy group were significantly smaller than those in the high-risk malignancy group. More than half of the SMTs were located in the gastric fundus and body.

**Table 1:** Baseline characteristics of the study population.

Variables	All patients (*n* = 189)	Non-GISTs (*n* = 79)	GISTs (*n* = 110)			*P* value
			Other (*n* = 26)	Low-risk (*n* = 46)	High-risk (*n* = 38)	
Age, yr	57.6 (11.57)	52.7 (11.26)	62.5 (8.88)	59.0 (10.87)	62.6 (10.92)	0.140
Sex						0.187
Male	92 (48.7)	48 (60.8)	9 (34.6)	16 (34.8)	19 (50.0)	
Female	97 (51.3)	31 (39.2)	17 (65.4)	30 (65.2)	19 (50.0)	
Lesion size						<0.001
<2 cm	81 (42.9)	43 (54.4)	7 (26.9)	27 (58.7)	4 (10.5)	
>2 cm	108 (57.1)	36 (45.6)	19 (73.1)	19 (41.3)	34 (89.5)	
Lesion location						0.725
Cardia	9 (4.8)	7 (8.9)	0 (0)	1 (2.2)	1 (2.6)	
Fundus	68 (36.0)	10 (12.7)	13 (50.0)	23 (50.0)	22 (57.9)	
Body	61 (32.3)	21 (26.6)	9 (34.6)	19 (41.3)	12 (31.6)	
Antrum	12 (6.3)	10 (12.7)	1 (3.8)	1 (2.2)	0 (0)	
Extragastric	39 (20.6)	31 (39.2)	3 (11.5)	2 (4.3)	3 (7.9)	

### Performance of lesion classification and segmentation

**Figure 2:**
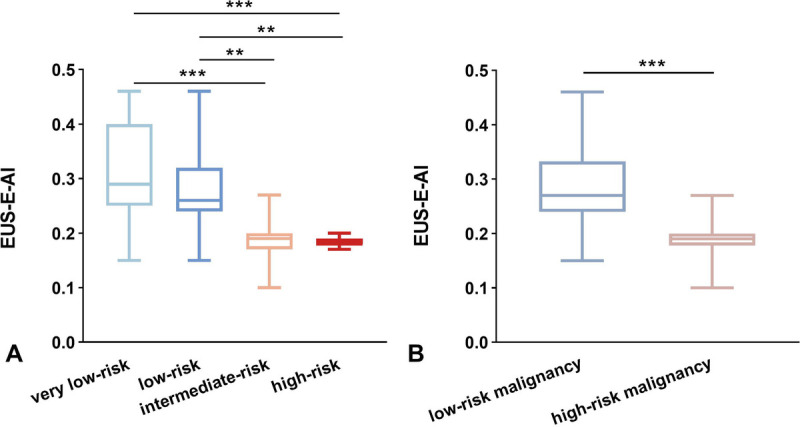
EUS-E-AI values of GIST patients. (A) Comparison of EUS-E-AI values between 4 risk groups according to NIH classification. (B) Comparison of EUS-E-AI values between low-risk malignancy group and high-risk malignancy group. ***P < 0.001, **P < 0.01.

DCNN1 achieved an accuracy of 95.8% in distinguishing GIST and non-GIST images, with a sensitivity of 96.7% and a specificity of 94.5% for GIST identification [Figure S3, http://links.lww.com/ENUS/A382]. The Dice coefficient for the segmentation model (DCNN2) was 0.967, with a recall and precision of 97.0% and 96.2%, respectively, at a 50% Intersection over Union (IoU) threshold. The EUS-E-AI values of GIST patients in the low-risk malignancy and high-risk malignancy groups were extracted and compared. The median EUS-E-AI value in the low-risk malignancy group (0.268 [IQR, 0.243–0.333]) was significantly higher than that in the high-risk malignancy group (0.186 [IQR, 0.176–0.199], *P* < 0.001) [Figure 2]. The correlation coefficient between EUS-E-AI and NIH risk classification for GISTs was −0.619, and the correlation coefficient with the malignant risk stratification was −0.624 [Table 2].

**Table 2:** Correlation between clinical-pathological characteristics and EUS-E-AI.

	EUS-E-AI	Lesion size	Malignant potential	NIH classification	Mitotic count
EUS-E-AI				
Coefficient	1.000	−0.291	−0.624	−0.619	−0.197
*P* value		0.007	<0.001	<0.001	0.072
Lesion size				
Coefficient		1.000	0.497	0.623	0.201
*P* value			<0.001	<0.001	0.067
Malignant potential				
Coefficient			1.000	0.908	0.495
*P* value				<0.001	<0.001
NIH classification				
Coefficient				1.000	0.537
*P* value					<0.001
Mitotic count				
Coefficient					1.000
*P* value				

### Performance of risk stratification

**Figure 3:**
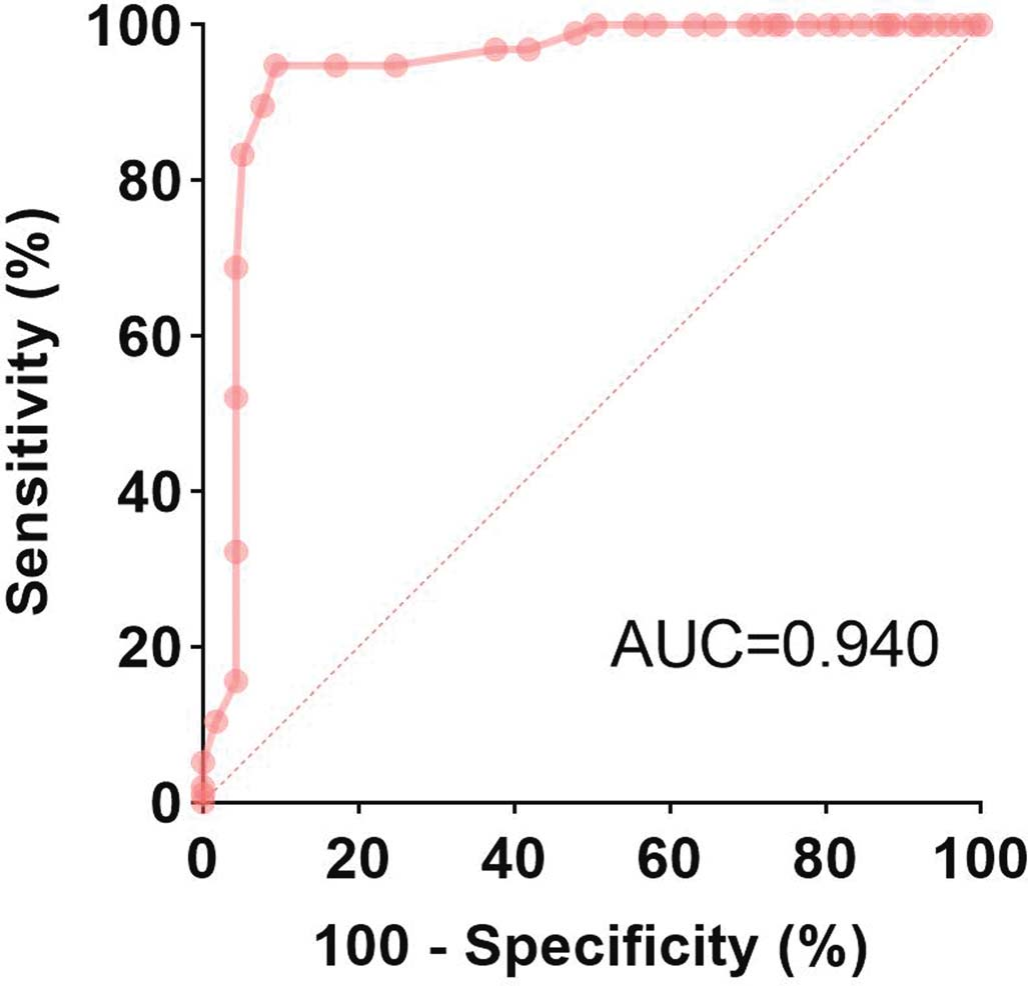
ROC curve of the EUS-E-AI value for the classification of malignant potential.

**Figure 4:**
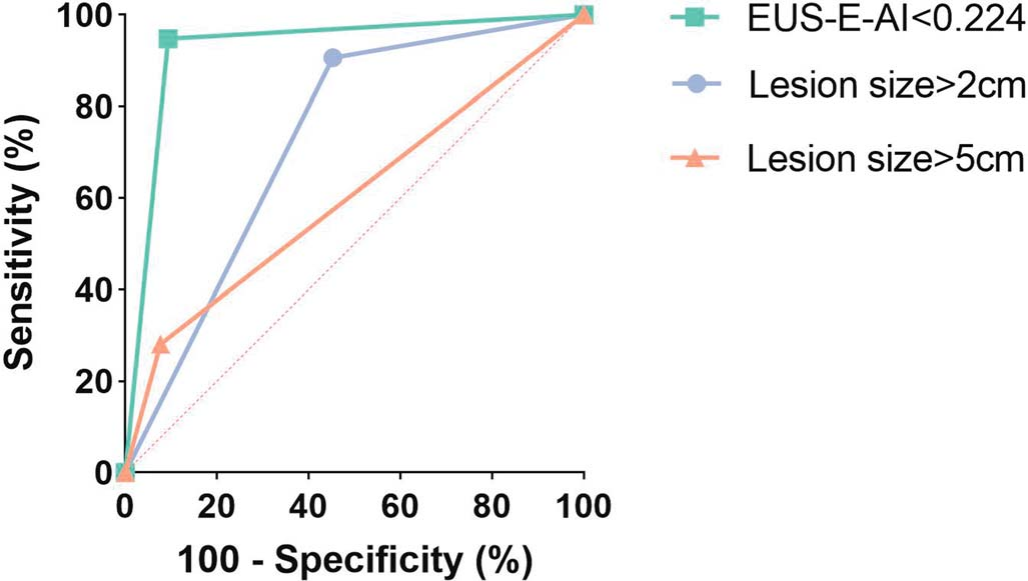
ROC curve of the cutoff EUS-E-AI (0.224) and lesion size for the classification of malignant potential.

In ROC analysis, the optimal cutoff value of EUS-E-AI for distinguishing low-risk and high-risk GISTs was 0.224 [Figure 3]. At this cutoff, the sensitivity for diagnosing high-risk malignant potential patients was 94.8% (95% CI, 90.3–99.2), specificity was 90.7% (95% CI, 85.4–96.0), positive predictive value was 89.2% (95% CI, 83.2–95.2), negative predictive value was 95.4% (95% CI, 91.5–99.2), and accuracy was 92.6% (95% CI, 89.1–96.1), with an area under the curve (AUC) of 0.940 (95% CI, 0.903–0.977). As shown in Figure 4, the AUC was higher than that of size-based criteria, such as lesions larger than 2 cm (AUC, 0.727; 95% CI, 0.658–0.795) or 5 cm (AUC, 0.602; 95% CI, 0.525–0.680). In both univariate and multivariate analyses, lesions larger than 2 cm (odds ratio, 10.331; 95% CI, 2.134–50.020) and EUS-E-AI less than 0.224 (odds ratio, 37.035; 95% CI, 8.736–156.999) were significant predictors for high-risk malignancy potential patients [Table 3].

**Table 3:** Logistic regression analyses for predictors of high-risk malignant potential GISTs.

	Univariable analysis		Multivariable analysis	
Variables	Odds ratio (95% CI)	*P* value	Odds ratio (95% CI)	*P* value
Age, yr	1.031 (0.990–1.075)	0.142	1.001 (0.931–1.076)	0.972
Sex	0.533 (0.221–1.284)	0.161	0.763 (0.187–3.117)	0.706
Location	1.235 (0.288–5.307)	0.776	0.942 (0.705–11.875)	0.963
Size >2 cm	12.079 (3.672–39.733)	<0.001	10.331 (2.134–50.020)	0.004
EUS-E-AI <0.224	40.375 (11.155–146.135)	<0.001	37.035 (8.736–156.999)	<0.001

### Subgroup analysis of GISTs <2 cm

A subgroup analysis was conducted on 31 GIST patients with lesions <2 cm, including 27 with low-risk malignancy and 4 with high-risk malignancy. In this subgroup, the median EUS-E-AI value in the low-risk malignancy group (0.283 [IQR, 0.246–0.387]) was significantly higher than that in the high-risk malignancy group (0.168 [IQR, 0.160–0.217], *P* < 0.001) [Figure S3, http://links.lww.com/ENUS/A382]. When the cutoff EUS-E-AI value of 0.224 was applied for differential diagnosis in this subgroup, the sensitivity, specificity, and accuracy were 75.0% (95% CI, 32.6–100.0), 81.5% (95% CI, 66.3–96.7), and 80.65% (95% CI, 66.5–94.8), respectively, with an ROC-AUC of 0.842 (95% CI, 0.667–1.000) (Figure S5, http://links.lww.com/ENUS/A382).

## DISCUSSION

GISTs are a type of tumor with malignant potential. Guidelines recommend regular follow-up for low malignant potential GISTs with a diameter less than 2 cm, whereas high malignancy potential GISTs should undergo surgical resection and preoperative targeted therapy to improve the complete resection rate.^[1,5]^ Therefore, preoperative risk stratification for GIST patients is crucial for clinical decisions. In this study, we developed an AI-based identification and risk stratification system for GISTs under EUS, which demonstrated satisfactory diagnostic performance in distinguishing GISTs from other SMTs. This system not only accurately segments the boundaries of GISTs but also objectively extracts the elasticity value (EUS-E-AI) from the lesion area under EUS-E. We further validated the potential utility of the EUS-E-AI in the stratification of GISTs' malignant potential. The results indicated that the EUS-E-AI value in the low-risk malignancy group was significantly higher than that in the high-risk malignancy group. Moreover, a cutoff value of 0.224 for the EUS-E-AI was found to effectively differentiate low-risk from high-risk malignancy group. This finding was also confirmed in GISTs with diameters smaller than 2 cm, indicating that the method is applicable to both large and small GISTs for risk stratification. Notably, factors such as patient age, sex, lesion size, and lesion location did not significantly affect the predictive ability of this cutoff value.

In recent years, several studies have explored the application of CT and MRI for prognostic prediction in GISTs. Yang et al.^[21]^ applied LASSO regression to predict individual preoperative risk for GIST patients using manually extracted MRI features. Lin et al.^[22]^ developed a mitotic prediction model and risk stratification nomogram for GISTs based on 4 CT radiologic features and clinical risk factors. Their nomogram showed comparable accuracy to pathological stratification (AUC: 0.965 *vs.* 0.983, *P* = 0.117). Despite excellent results, most existing radiomics models rely heavily on manually selected features, and their performance is limited by the lack of standardized solutions for optimizing key steps in feature extraction and classification, reducing their clinical applicability. Compared to CT and MRI, EUS provides more comprehensive information for the diagnosis of GISTs and is considered as the preferred method for assessing SMTs. Shah et al.^[23]^ confirmed that EUS features, such as irregular borders, local infiltration, and heterogeneity, are predictive factors for malignancy. Building on this, Li et al.^[24]^ developed an EUS-based model to differentiate high-risk from low-risk GIST patients, with an accuracy of 82.3%. However, this model is driven by experience or knowledge, and the assessment of EUS features is subjective, which may limit the model's predictive potential. Cai et al.^[19]^ used a machine learning algorithm to create the AORMS framework for automatic parameter selection, achieving an AUC of 0.750 for GIST risk stratification. Unfortunately, this study primarily focused on lesions smaller than 2 cm, and its applicability to larger GISTs remains uncertain.

Elastography is a real-time technique for tissue stiffness assessment, which reflects the elasticity of tissues and is closely related to the pathological characteristics of lesions. Malignant tumors, such as those caused by fibrosis, proliferated vascular or infiltrative malignancy, tend to have increased stiffness, resulting in abnormal elasticity patterns.^[25]^ EUS-E can complement B-mode EUS and neither adds additional risk nor significantly increases the examination time. It has been widely applied in the differential diagnosis of chronic pancreatitis and pancreatic solid tumors.^[13,15,26]^ Previous studies have shown that EUS-E shows high sensitivity and specificity in distinguishing GISTs from other SMTs, with sensitivity at 100% and specificity at 94.1%.^[16,27]^ However, the application of EUS-E in the risk stratification of GISTs has not yet been reported.

EUS-E was initially a qualitative technique, in which green areas correspond to soft tissue and are considered benign, whereas blue areas are more likely to indicate malignancy. However, this approach relies solely on the endoscopist's subjective judgment, which may introduce potential bias. Currently, the strain ratio (SR) is primarily used for semiquantitative analysis. In this method, the endoscopist selects a circular ROI over the lesion (A) and another smaller ROI (B) over a soft reference area to calculate the SR.^[28,29]^ Although this approach reduces bias in color assessment, it still involves some subjectivity, as the ROI selection remains operator-dependent. Moreover, the reference area is often chosen from the softest part of the gastric wall, but variations in size and location between the reference and lesion areas can significantly affect the measurement accuracy.^[30]^ This may also be one of the reasons why EUS-E has not been widely applied in GISTs. Minimizing interobserver variability and improving reproducibility remain key challenges for EUS-E.

AI has the potential to contribute to more objective and accurate analyses.^[31,32]^ In this study, the deep learning–based lesion classification and segmentation model enables automatic identification and boundary segmentation of GISTs, eliminating the need for manual ROI selection by the operator. This overcomes the limitations of traditional EUS scanning methods and reduces the bias associated with the ROI selection. Additionally, based on the lesion segmentation, we extracted the pixel values of all colored pixels within the lesion area and calculated the average pixel value to develop a novel elastography index, EUS-E-AI. This indicator comprehensively considers the overall characteristics of the lesion and quantifies the tissue stiffness, providing a more objective and accurate reflection of lesion stiffness, independent of the endoscopist's experience or subjective judgment. Our results revealed a significant correlation between EUS-E-AI and the NIH risk classification of GISTs (Spearman's correlation coefficient = −0.619, *P* < 0.001), effectively identifying 94.8% of high-risk malignant potential patients. This demonstrates that our system offers a reliable basis for risk stratification, providing substantial clinical application value for clinicians.

The management of GISTs smaller than 2 cm remains controversial, as there is insufficient evidence supporting the benefit of surgical resection for these tumors.^[5]^ However, surgical resection is still the recommended treatment for small GISTs with high malignant potential. To address this, we conducted a subgroup analysis of GIST patients with sizes smaller than 2 cm and evaluated the effectiveness of EUS-E-AI in risk stratification. Among 31 patients with small GISTs, the EUS-E-AI cutoff value of 0.224 demonstrated an accuracy of 80.65% in distinguishing low-risk from high-risk malignancy, a result comparable to that of Cai et al. These findings suggest that EUS-E-AI can serve as a valuable tool for assessing the malignant potential of small GISTs. Furthermore, this system may be particularly helpful in the management of borderline-sized GISTs (approximately 2 cm), where current clinical guidelines are less definitive and the choice between surveillance and local resection remains controversial. By providing a quantitative and objective measure of lesion stiffness, EUS-E-AI may support more individualized treatment decisions in this patient population.

Some limitations of this study should be acknowledged. First, because this is a retrospective study, potential selection biases are inevitable. Thus further validation of EUS-E-AI should be conducted in prospective cohorts, and we are currently carrying out such research. Second, the number of GIST patients with tumors smaller than 2 cm was limited in this study. Future studies should increase the sample size and recruit more patients with small GISTs to better evaluate the application of EUS-E-AI in this subgroup. Notably, the subgroup of high-risk GISTs <2 cm in our study included only 4 cases, which limits statistical representativeness. Therefore, these results should be interpreted with caution, and further validation in larger prospective cohorts is warranted.

Additionally, the elastography technique used in this study was real-time strain elastography, a qualitative method based on tissue deformation under manual compression. In contrast, shear wave elastography is a more recent, quantitative technique that measures tissue stiffness via shear wave propagation velocity. This method may offer improved reproducibility and operator independence. Future prospective studies are warranted to explore the clinical value of shear wave elastography in GISTs and to compare its performance with strain elastography and AI-based approaches such as EUS-E-AI. Such comparative analyses could further clarify the role of elastography in preoperative risk stratification and clinical decision-making.

In conclusion, we developed a deep learning–based indicator, EUS-E-AI, for the objective assessment of lesion stiffness, aimed at assisting clinicians in preoperative risk stratification of GISTs. This tool may contribute to the differential diagnosis, treatment decision-making, and follow-up planning for GIST patients.

## Ethical Approval

The study was approved by the Ethics Committee of Renmin Hospital of Wuhan University.

## Informed Consent

Given its retrospective design, the requirement for written informed consent was waived.

## Source of Funding

This study was funded by Key Research and Development Program of Hubei Province (grant no. 2023BCB153, to Honggang Yu), Project of Hubei Provincial Clinical Research Center for Digestive Disease Minimally Invasive Incision (grant no. 2024CCB007, to Honggang Yu), and National Key R&D Program of China (grant no. 2022YFC2505105, to Wei Zhou).

## Conflicts of Interest

All authors declare no conflicts of interest.

## Author Contributions

Honggang Yu and Wei Zhou conceived and designed the study; Chenxia Zhang, Wei Tan, and Xun Li collected the data; Chenxia Zhang, Wei Tan, Xiao Tao, BX, and Xun Li analyzed the data, Chenxia Zhang and Xun Li wrote the manuscript; all authors reviewed and approved the final manuscript for submission.

## Data Availability Statement

The data that support the findings of this study after deidentification are available from the corresponding author upon reasonable request.
